# Shifting the Specificity of *E. coli* Biosensor from Inorganic Arsenic to Phenylarsine Oxide through Genetic Engineering

**DOI:** 10.3390/s20113093

**Published:** 2020-05-30

**Authors:** Hyojin Kim, Yangwon Jeon, Woonwoo Lee, Geupil Jang, Youngdae Yoon

**Affiliations:** 1 Department of Environmental Health Science, Konkuk University, Seoul 05029, Korea; gywls6772@konkuk.ac.kr (H.K.); ywjun69@konkuk.ac.kr (Y.J.); lunia2005@hanmail.net (W.L.); 2 School of Biological Sciences and Technology, Chonnam National University, Gwangju 61186, Korea; yk3@jnu.ac.kr

**Keywords:** organic arsenic, arsenic-responsive operon, ArsR, protein engineering, *E. coli*-based biosensors

## Abstract

It has recently been discovered that organic and inorganic arsenics could be detrimental to human health. Although organic arsenic is less toxic than inorganic arsenic, it could form inorganic arsenic through chemical and biological processes in environmental systems. In this regard, the availability of tools for detecting organic arsenic species would be beneficial. Because As-sensing biosensors employing arsenic responsive genetic systems are regulated by ArsR which detects arsenics, the target selectivity of biosensors could be obtained by modulating the selectivity of ArsR. In this study, we demonstrated a shift in the specificity of *E. coli* cell-based biosensors from the detection of inorganic arsenic to that of organic arsenic, specifically phenylarsine oxide (PAO), through the genetic engineering of ArsR. By modulating the number and location of cysteines forming coordinate covalent bonds with arsenic species, an *E. coli* cell-based biosensor that was specific to PAO was obtained. Despite its restriction to PAO at the moment, it offers invaluable evidence of the potential to generate new biosensors for sensing organic arsenic species through the genetic engineering of ArsR.

## 1. Introduction

Heavy metals and metalloids, including arsenics, in environmental systems are typically quantified via an instrument-based analysis that is limited by several shortcomings such as expensive and time-consuming processes. As alternatives, diverse sensors for monitoring metal(loid) easily and speedily—including electrochemical technique-based sensors, chemical compound-based sensors, metal binding protein coupled with fluorescent probe-based sensors and microorganism cell-based sensors—have been developed and reported [1,2,3,4]. Among these new techniques, living cell-based sensors, referred to as whole-cell bioreporters (WCBs), that use their own metal-responsive genetic systems have been extensively investigated as they are relatively uncomplicated and cheap [5,6]. Additionally, this technique has been highlighted by its capability to measure bioavailability.

The WCBs consist of sensing and reporter domains. Sensing domains recognize targets and initiate the transcription of reporter genes while reporter domains represent and indicate the presence and concentration of targets through differentiated expression [7,8]. For example, znt- and ars-operons in *E. coli* regulate the transcription of genes under promoters upon the interaction of regulatory proteins named ZntR and ArsR with Zn and As ions, respectively [9,10]. However, the number of WCBs that could detect environmental pollutants is limited by the number of genetic systems that are usable in WCBs. In this regard, the generation of new WCBs from the available genetic systems and identification of new genetic systems would be invaluable. In fact, substantial efforts to generate new WCBs that are specific to new targets from known genetic systems using genetic and biochemical engineering have been made [11,12,13,14].

Among the diverse heavy metal(loid)s, arsenic is a well-known hazardous metalloid due to its strong toxicity to living organisms, and a ubiquitous environmental carcinogen that is linked to diverse types of cancers such as skin cancer [15,16]. Although the toxicity of inorganic arsenic is regarded as a major concern, diverse organic arsenic compounds, such as methylarsenate and phenylarsenite, are widely used as herbicides and pesticides [17,18,19]. The toxic effects of organic arsenic have been found to be less severe than those of inorganic arsenic. However, organic arsenic is severely toxic to living organisms and is a potential source of inorganic arsenic in environmental systems [20,21]. Thus, organic arsenic compounds in environmental systems should be taken into consideration in monitoring and control initiatives. Unfortunately, a genetic system that is specifically usable in WCBs for organic arsenic is unavailable, necessitating investigation through instrument-based analysis [22,23].

The arsenic responsive genetic systems, called *ars*-operons, in microorganisms have been employed as domains for detecting arsenic species. The ArsR, a regulatory protein, plays an essential role in identifying arsenic ions including As (III) and As (V). Additionally, it exhibits an affinity to antimony (Sb) and some organic arsenic compounds. Consequently, the WCBs based on ars-operon systems detect a broad range of targets in the following order of strength: As (III) > As (V) > Sb (III) > organic arsenic compounds [24]. The interaction of ArsR with targets is dependent on the coordination of cysteine residues in binding sites, and would thus be modulated by rearranging amino acids in ArsR interacting with targets. There have been several reports on WCBs specifically detecting organic arsenics such as methyl and aromatic arsenics through the genetic engineering of ArsR [19,25,26]. In these studies, the target selectivity of ArsR was changed to organic arsenics such as methylarsenate and phenylarsenite by deleting single cysteine making coordination number less. However, a large number of organic arsenics are continually produced and released into environmental systems, making the aforementioned detections insufficient. In this regard, a systematic approach towards the development of biosensors targeting organic arsenics is necessary. In this study, we developed of a phenylarsine oxide (PAO) specific biosensor and demonstrated the potential to generate new biosensors for detecting organic arsenic species using genetic engineering.

## 2. Materials and Methods

### 2.1. Materials

*Escherichia coli* (*E. coli*) were used as competent cells for cloning and as host cells for biosensors. The endogenous *arsR* gene in *E. coli* encoding ArsR, the regulatory protein, was deleted using the Quick & Easy *E. coli* Gene Deletion Kit (Gene Bridges; Heidelberg, Germany) to generate a mutant strain called *E. coli* (DH5 α)-arsR (Δ*arsR::kan*). The plasmids bearing recombinant *arsR* WT and mutants, as well as the fusion of the promoter of *ars*-operon and *egfp* encoding enhance green fluorescence protein (eGFP) used in this study were generated from our previous study. The arsenic species such as AsCl_3_, HAsNa_2_O_4_Cl and PAO were purchased from Sigma-Aldrich (St. Louis, MO, USA) and prepared as 1 mM stocks by being dissolved in adequate solution. Fluorescence spectrophotometer (Scinco, Seoul, Korea) equipped with a xenon lamp as a light source (300–800 nm excitation/emission wavelength) and a set of 0.1–5 nm bandwidth filters were used to measure fluorescence signals.

### 2.2. E. coli-Based Biosensors for Arsenic Species

The gene encoding regulatory protein, ArsR (GenBank no. AVD50083.1), was obtained by PCR from genomic DNA of *E. coli* and inserted into expression vector, pCDF-Duet. The *E. coli*-based biosensors were generated by inserting a set of plasmids carrying arsR WT and mutants, pCDF-ArsR WT and mutants, and *arsR* promoter region fused with *egfp*, pArs-eGFP. A set of plasmids were co-transformed into *E. coli* (DH5 α)-arsR and transformants with both plasmids were screened on a Luria Broth (LB) plate with spectinomycin and ampicillin. Overall, five biosensors were generated and tested in this study, as listed in Table 1.

### 2.3. Biosensor Assays for Selectivity and Specificity

The overnight culture of *E. coli* (DH5α)-arsR cells harboring two plasmids carrying *arsR* and *egfp* regarded as sensing and reporter domains, respectively, were inoculated in fresh LB supplemented with spec/amp and incubated at 37 °C in a shaking incubator until the value of optical density at 600 nm (OD_600_) reached 0.4. The incubated cells were divided into 5 mL samples and exposed to arsenic species including As (III), As (V) and PAO with concentration ranging from 0 to 5 μM. To evaluate the toxic effects of arsenic species, the cells exposed to arsenic species were incubated for 4 h as OD_600_ values were measured. The biosensor assays were performed according to the protocol used in our previous study with minor modification [27]. Briefly, the fluorescence intensity from *E. coli* biosensors induced by arsenic species was measured using FC-2 spectrophotometers upon 1 and 2 h of incubation. The fluorescent signals were determined at a range of 500 to 600 nm of emission with 480 nm of excitation wavelength. The bandwidths of both excitation and emission filters were set as 5 nm. The arbitrary fluorescent signals were converted to induction coefficient values, which was expressed as [fluorescence intensity of biosensors exposed to arsenics]/[fluorescence intensity of non-exposed biosensors] to minimize the errors.

### 2.4. Quantification of PAO in Artificially Contaminated Water Samples

To verify applicability, the samples containing a known concentration of PAO for standard curves and artificially contaminated water samples were prepared by spiking PAO to certain levels of concentrations. The PAO-specific biosensors possessing ArsR-C37S/L36C as regulatory protein were exposed to a 0 to 1.5 μM range of PAO and the dilutions of artificially contaminated samples. After 1 h of exposure, the responses of biosensors were indicated as induction coefficient values and plotted against the concentration of PAO to obtain standard curves. Based on the equation fitted to the standard curve, the concentration levels of PAO in contaminated samples were calculated and the results are summarized in Table 2. The accuracy of the biosensor assay was indicated as a percentage of [calculated concentration of PAO]/[spiked concentration of PAO].

### 2.5. Homology Modelling

The SWISS-MODEL server was used to build model structures [28,29]. The server identified fifty templates that showed high sequence similarity, and showed that the 3D structure that was most similar to the sequence of ArsR was cyanobacterial metallothionein repressor SmtB (1 smt.pdb) as 39.76% of sequence identity. The model structures of ArsR and its mutant were built based on the structure of SmtB and subjected to energy minimization using Sybyl software (Tripos, St. Louis, MO, USA). All structures were visualized and examined using UCSF Chimera software [30].

## 3. Results

### 3.1. Toxic Effects of Arsenic Species on the Growth of E. coli

**Figure 1:**
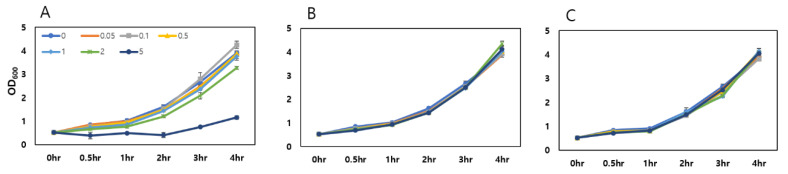
Toxic effects of arsenic species on *E. coli* biosensors. PAO (**A**), As (III) (**B**) and As (V) (**C**) on the growth of *E. coli*. 0 to 5 μM of arsenic species were treated to *E. coli* cells when the OD_600_ values were reached to 0.5, and OD_600_ values were determined during 4 h. The experiments were repeated more than thrice and the standard deviation was represented by error bars.

As the arsenic species are toxic to living organisms, the establishment of toxic effects on the host cell of biosensors is necessary. In order to measure toxicity, the biosensor host cell *E. coli* DH5α-*arsR*, was exposed to 0 to 5 μM range of As (III) and As (V), and PAO. The cells were exposed to arsenic species when the OD_600_ reached 0.5 and the OD_600_ values were continuously measured during the 4 h period. As shown in Figure 1, growth inhibition was only observed over 2.0 μM of PAO, while inorganic arsenic species showed no effect on growth at given concentration ranges. Interestingly, the toxic effects of PAO on the growth of *E. coli* were significantly stronger than those of As (III) and As (V) because organic arsenics are normally relatively less toxic. Based on this result, the concentration ranges of arsenic species for biosensor assay should be adjusted because higher toxic effects yielded inaccurate results. Thus, we further investigated biosensor assays with 0 to 1 μM ranges of arsenic species to eliminate errors caused by the toxicity of targets on biosensor cells.

### 3.2. Specificity of ArsR WT toward Arsenic Species

**Figure 2:**
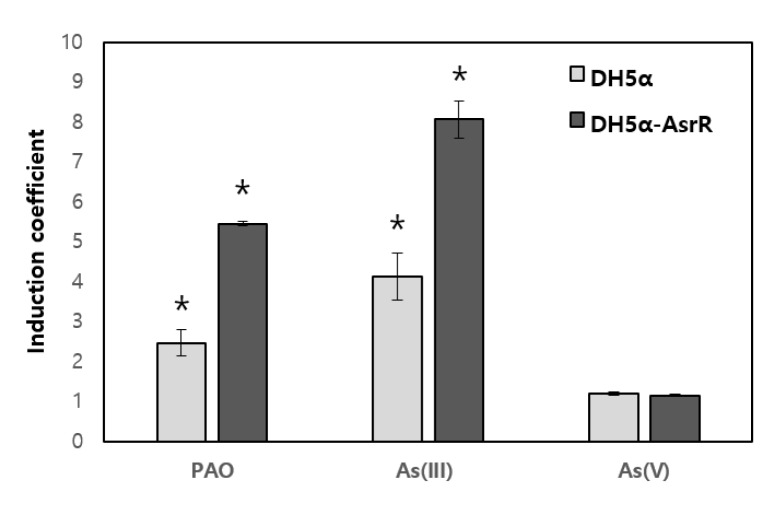
Selectivity of ArsR WT toward arsenic species. The responses of biosensors possessing endogenous ArsR WT (DH5α) and recombinant ArsR WT (DH5α-*arsR*) with pArs-eGFP toward 1.0 μM of PAO, As (III) and As (V) were indicated using light and dark grey bars, respectively. The error bars were represented as standard deviation. All the values showed significant differences compared to the value without arsenic species exposure were indicated as asterisks (*) (*p* > 0.05). The experiments were repeated more than three times and the standard deviation was represented by error bars.

The sensing capability of *E. coli*-based biosensors is determined by regulatory proteins, especially ArsR in this study. To verify the target recognition of ArsR WT, 1.0 μM As (III), As(V) and PAO were treated to two *E. coli* biosensors, WT *E. coli* DH5α with pArs-eGFP and *E. coli* DH5α-arsR with pArs-eGFP and pCDF-ArsR WT. The former has endogenous ArsR WT and the latter has recombinant ArsR WT. The responses of biosensors toward each arsenic species were represented as induction coefficient values (Figure 2). As shown in Figure 2, the response to As (III) was highest indicating that ArsR WT interacts most with As (III). PAO ranked second with respect to the strength of responses and As (V) was the weakest. Although the induction coefficient value toward each arsenic species was different from each biosensor, the patterns were similar. This may have been attributable to the difference in the number of ArsR molecules in cells owing to the expression level of the endogenous and recombinant genes. In particular, it was confirmed that ArsR WT exhibits broad specificity toward three arsenic species with varied affinity.

### 3.3. Modulating Selectivity to Arsenic Species by Rearranging Cysteines in ArsR

**Figure 3:**
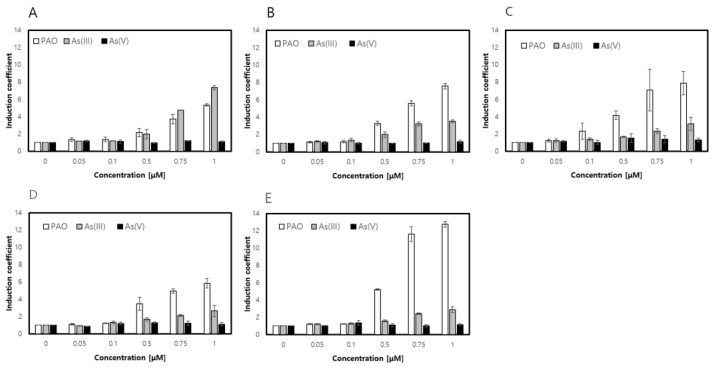
Modulation of specificity toward arsenic species upon ArsR mutation. The induction coefficient values of five biosensors, ArsR WT (**A**), ArsR-C37S (**B**), ArsR-C37S/T38C (**C**), ArsR-C37S/L39C (**D**) and ArsR-C37S/A36C (**E**), were exposed to PAO, As (III) and As(V) ranging from 0 to 1 μM. The experiments were repeated more than thrice and the standard deviation was represented using error bars.

The gene encoding ArsR was amplified from genomic DNA of in *E. coli* DH5α in our previous studies and inserted into modified pCDF-Duet vector possessing P_tac_ promoter instead of T7 [24]. The arsenic binding site of ArsR has three cysteines, Cys32, Cys34 and Cys37 known to form coordination bonds with arsenic ions. In this regard, the mutations and rearrangement of cysteines in the arsenic binding site of ArsR were conducted to modulate the specificity of ArsR, resulting in three classes of ArsR mutants. Among them, two classes of mutants with Cys32Ser and Cys34Ser showed no significant effects except attenuation in their responses to arsenic species (data not shown). On the contrary, the mutants with Cys37Ser showed differentiated responses toward As (III), As (V) and PAO (Figure 3). ArsR-C37S showed a similar level of responses to PAO and As (V) to ArsR WT, and significantly weaker responses to As (III) (Figure 3A,B). With additional mutations on ArsR-C37S including relocation of cysteines on L36, T38 and A39, the responses to PAO and As (III) varied significantly. The responses of ArsR-C37S/T38C and ArsR-C37S/A39C to PAO were similar but were significantly weaker to As (III) compared to WT (Figure 3C,D). Interestingly, the response to PAO was doubled in ArsR-C37S/L36C while the response to inorganic arsenics including As (III) and As (V) was relatively weaker (Figure 3E). The disruption caused by the formation of co-ordinates arsenic ions with ArsR weakened the interaction with arsenic ions by deleting cysteines and the relocated cysteines enhanced the interaction with PAO, which explains the aforementioned result. This answer is obviously not comprehensive, but it was inferred that the conformation changes by mutations altered the selectivity of ArsR to PAO rather than to inorganic arsenics. Additionally, the biosensors with ArsR-C37S/T38C and C37S/L36C were found to be highly specific to PAO, making them potential PAO-specific biosensors.

### 3.4. PAO-Specific E. coli Cell-Based Biosensor

**Figure 4:**
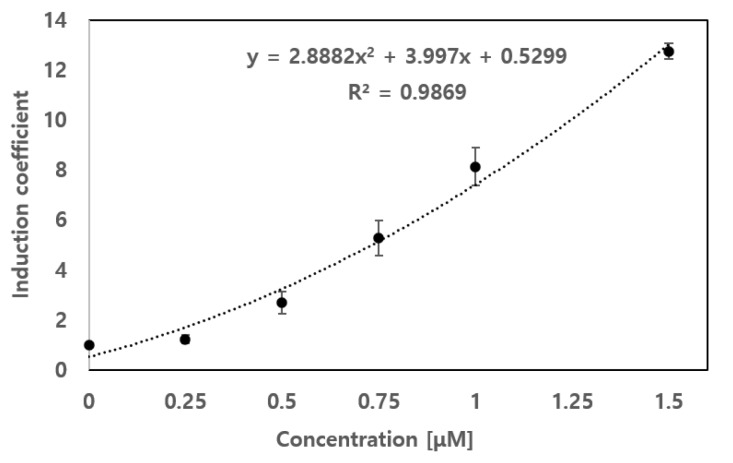
Standard curve for PAO from *E. coli* biosensor with ArsR-C37S/L36C. The induction coefficient values were plotted against a 0 to 1.5 μM range of PAO. The standard curve fit to correlation was obtained and the equation was used to quantify PAO in artificially contaminated water samples. The experiments were repeated more than thrice and the standard deviation was represented by error bars.

As *E. coli* cell-based biosensors harboring ArsR-C37S/L36C and ArsR-C37S/A39C showed enhanced selectivity to PAO and decreased selectivity to inorganic arsenic compared to ArsR WT, the applicability for quantification of PAO was further investigated. However, compared to ArsR-C37S/A39C in terms of relative responses to PAO and As (III), the biosensor possessing ArsR-C37S/L36C showed superior specificity toward PAO. The former PAO and As (III) ratio of more than 4 then later became a ratio of approximately 2 (Figure 3D,E). In this regard, the biosensor possessing ArsR-C37S/L36C as regulatory proteins was selected as a candidate for detecting PAO. To verify the applicability of the new biosensor in monitoring PAO levels in environmental samples, it was exposed to a range of 0 to 1.5 μM of PAO. The responses of the biosensors were indicated as induction coefficient values and plotted against the concentration of PAO to obtain a standard curve. At the same time, biosensors were exposed to the artificially contaminated samples with different dilutions under the same experimental conditions and determined the induction coefficient values. As shown in Figure 4, the induction coefficient values of biosensors to PAO was increased as a concentration dependent manner and fit to quadratic function with 0.987 of R square values. Based on the equation of standard curves, the concentration of PAO in artificially contaminated samples containing 0.3, 0.9, and 1.2 μM of PAO were determined as 0.313, 0.999, and 1.155 μM, respectively. The results are summarized in Table 2 and the accuracy of the biosensor assay exceeded 90%. Therefore, the new biosensor with ArsR-C37S/L36C could be applied as a PAO-specific biosensor.

### 3.5. Computational Structural Analysis for ArsR and Mutants

To build 3D structures of ArsR and its mutants, templates preliminarily from the SWISS-MODEL server were selected. Among the fifty templates for ArsR and its mutants, cyanobacterial metallothionein repressor SmtB showed the highest sequence identity: 39.76% for ArsR wild type, ArsR-C37S and ArsR-C37S/T38C and 38.55% for ArsR-C37SL39C and ArsR-C37S/A36C. The SmtB structure was therefore selected for the template and built model structures. All model structures were validated with Qualitative Model Energy analysis (QMEAN) values and PROCHECK. Any phi and psi angles in the disallowed region were not found after the PROCHECK analysis. The QMEAN values represent the quality of model structure estimation calculated on the basis of both local and global geometry. The values that are close to zero were good, while those below −4 were unacceptable. The QMEAN values of our model structures varied from −2.03 to −2.50, meaning they are reliable for further structure analysis.

**Figure 5:**
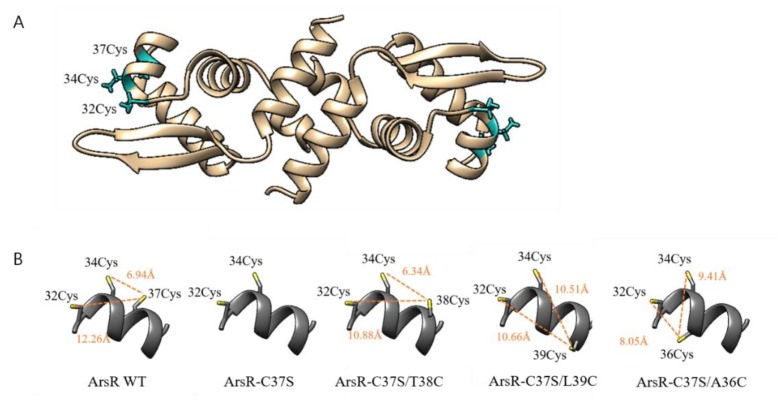
Structural analysis of ArsR WT and mutants. (**A**) 3-dimensional structure of ArsR dimer constructed by homology modeling with three cysteines in arsenic binding sites (green), (**B**) the location and distances between cysteines in arsenic binding sites of ArsR and mutants. The distances between sulfurs of cysteines were indicated, and the distances between 32Cys and 34Cys were within the range of 6.99 Å to 7.08 Å.

As shown in Figure 5, ArsR formed a homodimer and three cysteines were closely located in the arsenic binding sites of each monomer. The structures of ArsR mutants were also constructed based on the ArsR WT structure and minimization to obtain stable structures was performed. The cysteines on arsenic binding sites were highlighted as sticks and the distances and orientation of cysteines were displayed (Figure 5B). Although the structures of mutants were slightly different, the distances between sulfurs of between Cys32 and Cys34 ranged between 6.99 Å and 7.08 Å. The cysteines in ArsR WT formed a triangle fit to the As (III) ion best among three tested arsenic species. Interestingly, all other mutants showed inverse responses to As (III) and PAO when ArsR lost Cys37 (Figure 3). When cysteine was relocated, the responses toward PAO were increased while As (III) responses were decreased. Based on model structures, it was inferred the target selectivity of ArsR and the distances among cysteines would be related. The interaction between cysteines (except Cys37) and ArsR and As (III) was interrupted, while that between Cys38 and Cys36 and PAO was enhanced. Especially, ArsR-C37S/A36C forming smallest triangle showed superior response to PAO with diminished response to As (III). This computation approach showed the potential to modulate the target specificity of regulatory proteins as well as provided a reasonable explanation on how mutation varied with the target specificity of ArsR.

## 4. Discussion

Despite awareness of the harmfulness of organic arsenics, they have drawn less attention compared to inorganic arsenic species, owing to their relatively less toxicity. However, organic arsenics cannot be ignored because they have the potential to transform into toxic inorganic arsenic through diverse biochemical processes by living organisms [31,32,33]. Thus far, the approaches towards monitoring arsenic species have focused on inorganic arsenic. In particular, bacterial cell-based biosensors have been actively investigated following identification of the arsenic responsive genetic systems. The arsenic responsive genetic systems are controlled by ArsR which is known to recognize inorganic arsenic species with three cysteine residues [34]. Additionally, the coordinates with As ions induce conformational change, thus initiating the transcription as a response to As ions. Consequently, the target recognition of ArsR determines the target specificity of bacterial cell-based biosensors employing arsenic responsive genetic systems. In the same aspect, the PAO-specific biosensor could be generated by modulating the target specificity of ArsR.

The *E. coli* cell-based biosensors quantify the level of targets by measuring the expression level of reporter genes, and the expression level could be compared from the same number of cells. As the WCBs employed ArsR and eGFP as sensing and reporter domain, respectively, the portion of arsenic ions were determined by the expression level of eGFP. To eliminate the toxic effect of arsenic species on *E. coli*, the expression level (intensity) of eGFP was normalized by optical density values measured at 600 nm (OD_600_). Therefore, the toxic effects of As (III) and organic arsenic compound, PAO, were first investigated in this study (Figure 1). Unlike other organic arsenics such as methylarsenite (MAs (III)) roxarsone (Rox (III)) considered as being less toxic than inorganic arsenics, PAO showed stronger toxic effects on the growth of *E. coli*. However, this was expected because the adverse effects of PAO had been reported previously; it especially inhibits enzymatic activity of tyrosine kinase in T cells and endocytosis [35,36]. Although the environmental contamination of PAO had not been reported yet, it would at least be a reason to fabricate PAO sensing tools.

Even though the biosensor based on arsenic responsive genetic system had been used for inorganic arsenic sensing, it showed broad specificity toward As (III), As (V), Sb (III) and other organic arsenics. As shown in Figure 2, the biosensor with ArsR WT responded to As (III), As (V) and PAO. It was noticed the responses toward tested arsenic species were ordered as As (III) > PAO > As(V). This result was reasonable in the consideration of coordinate numbers. Since ArsR is known to interact with As (III) via coordinates with three cysteines, trivalent state of As (III) and PAO are preferred as binding partners. However, the three-dimensional coordination should be considered. In this regard, we believed the deletion and relocation of cysteines could modulate three-dimensional coordination, resulting in target selectivity.

Based on this notion, Cys37 was replaced by Ser and relocated cysteine on Leu36, Thr38, and Ala39 to modulate three-dimensional coordination and the coordination numbers of the arsenic binding site of ArsR. As shown in Figure 3, the selectivity of ArsR mutants changed by deleting Cys37 and relocating cysteines on distinct positions. Simply, it was assumed that the mutation of C37S decreases the affinity of ArsR to As (III) because the coordination number was decreased to 2. According to previous reports on organic arsenic sensing biosensors, the shift of specificity from As (III) to MA s(III), PhAs (III) or Rox (III) was caused by reducing the coordination number by mutating one cysteine of ArsR [25,26]. However, solely focusing on the coordination numbers would not satisfactorily explain this. It would sound reasonable but the bulkiness and steric hindrances between arsenics and ArsR were ignored. This notion was supported by following experimental results from cysteine relocation mutants including ArsR-C37S/T38C, -C37S/A39C and -C37S/L36C. It was clear that two coordination of ArsR-C37S lost affinity to As (III), but the other mutants, C37S/T38C, C37S/A39C and C37S/L36C, having three co-ordinations through cysteine relocation showed strong affinity toward only PAO not As (III). Additionally, responses to PAO and As (III) differed despite the cysteine relocated mutants of ArsR having three co-ordinates with three cysteines. This result suggested the significance of the three-dimensional coordination and the number of coordination and would be a signal to modulate the target specificity of regulatory proteins.

To elucidate the notion proposed in this study, three-dimensional structures of ArsR WT and mutants were constructed and cysteines in arsenic binding sites were analyzed. Clearly, the model structures were not real and therefore could not yield errors. However, they offered reasonable explanations and the results matched our observation. Although the number of cysteines in cysteine relocated mutants was similar to that of ArsR WT, the length and coordination were differentiated upon mutants (Figure 5B). To correlate the cysteines in binding sites and target specificity, As (III) was fit to only ArsR WT having Cys37 with 6.94 Å away from Cys34, while PAO was preferred shorter distance between Cys34 and third cysteine (Figure 5B). When the distance was increased beyond WT, i.e., ArsR-C37S/L39C, the response to PAO was even weaker than that of ArsR-C37S with two cysteines (Figure 3). Conclusively, the results could prove that the number of coordinates and spatial conformation should both be considered in the modulation of the target specificity.

The modulation of metal(loid) selectivity via the genetic engineering of regulatory proteins such as ZntR and ArsR has been reported in past studies [19,25,27]. For the arsenic species, the genetic engineering on ArsR made a shift in selectivity from As (III) to MAs (III) and PhAs (III) by changing the coordination numbers. This was the first study to generate a PAO-specific biosensor derived from ArsR engineering, and prove that the new biosensor could quantify PAO in environmental samples with over 90% accuracy (Figure 5 and Table 2). We believe that the biosensor derived from ArsR can be PAO-specific and practically applicable to environmental systems. Nonetheless, it was necessary to conduct functional validation of biosensor assay under field condition for practical applications because the environmental systems are far more complicated compared to laboratory conditions. We believed that the bacterial cell-based biosensors would be alternative tools to compensate rather than to replace analytical instruments. Overall, we demonstrated the generation of PAO-specific biosensors by genetic engineering on ArsR and explained the modulation of specificity by computational structural analysis. In fact, only PAO was available as non-inorganic arsenic species in this study because other organic arsenics were not allowed in the laboratory. Nonetheless, we believe that the practical application of the PAO-specific biosensor to environmental systems was worthwhile. In particular, the approach demonstrated in this study would be invaluable in the generation of new target sensing biosensors with limited genetic systems and multi-target sensing caused by the broad selectivity of regulatory proteins.

## Figures and Tables

**Table 1:** List of *E. coli* cell-based biosensors used in this study.

Host Cell	Reporter Domain	Sensing Domain
*E. coli* DH5α	pArs-eGFP	Endogenous ArsR WT
*E. coli* DH5α deleting *arsR*	pArs-eGFP	pCDF-ArsR WT
(*E. coli* DH5α-*arsR*)		pCDF-ArsR C37S
		pCDF-ArsR C37S/T38C
		pCDF-ArsR C37S/A39C
		pCDF-ArsR C37S/L36C

**Table 2:** Quantification of PAO in artificially contaminated water samples using *E. coli* biosensor with ArsR-C37S/L36C.

Spiked Concentration [μM]	Determined Concentration [μM]	Accuracy (%)
0.3	0.314 ± 0.016	95.7
0.9	0.999 ± 0.067	90.1
1.2	1.155 ± 0.095	96.2
